# Preventive interventions in individuals at risk for Rheumatoid Arthritis: State of the art and perspectives

**DOI:** 10.1016/j.jbspin.2023.105543

**Published:** 2023-02-15

**Authors:** Annette H.M. Van der Helm–van Mil

**Affiliations:** Department of Rheumatology, Leiden University Medical Center, PO Box 9600, 2300 RC Leiden, The Netherlands

**Keywords:** Rheumatoid arthritis, Prevention, Clinically suspect arthralgia, Subclinical joint inflammation

## Abstract

During the last decade, the outlook for patients with rheumatoid arthritis (RA) has improved greatly, especially for patients with autoantibody-positive RA. To further improve long-term disease outcomes, the field has turned to investigating the efficacy of treatment initiated in the pre-arthritic phase of RA, based on the adage “the sooner the better.” In this review, the concept of prevention is evaluated and different risk stages are being examined for their pre-test risks of RA development. These risks affect the post-test risk of biomarkers used at these stages and, consequently, the accuracy with which the risk of RA can be estimated. Furthermore, through their effect on accurate risk stratification, these pre-test risks ultimately also associate with the likelihood of false-negative trial results (the so-called “clinicostatistical tragedy”). Outcome measures to assess preventive effects are evaluated and relate to either the occurrence of disease itself or to the severity of risk factors for RA development. Results of recently completed prevention studies are discussed in the light of these theoretical considerations. The results vary, but clear prevention of RA has not been demonstrated. While some treatments (e.g. methotrexate) persistently reduced symptom severity, physical disability, and the severity of imaging joint inflammation, other treatments were not reported to produce long-lasting effects (hydroxychloroquine, rituximab, atorvastatin). The review concludes with future perspectives regarding the design of new prevention studies and considerations and requirements before findings can be implemented in daily practice in individuals at risk of RA attending rheumatology practices.

## 1 Introduction

During the last decades the prospects for newly diagnosed patients with rheumatoid arthritis (RA) have considerably improved. Debilitating joint destruction has become rare, the severity of functional impairment during the disease course has decreased, and DMARD-free remission has become within reach for some patients. These advances are attributed to earlier treatment initiation and treat to target treatment strategies, which have resulted in better suppression of joint inflammation compared to several decades ago. A recent study in RA with data of 25 years demonstrated that the improvement in long-term disease outcomes only apply to autoantibody-positive RA and not autoantibody-negative RA [1]. Autoantibody-negative RA patients currently experience a similar burden of disease as autoantibody-positive RA patients [2].

In terms of progress, autoantibody-positive RA remains a neglected patient group. Nonetheless, with the aim to further improve the disease burden the field is moving towards preventive interventions, initiated in individuals at-risk for RA but in whom clinically apparent arthritis has not occurred and a diagnosis of RA has not yet been made. Since the results of several intervention trials in individuals at risk for RA have recently been presented, this article will present the current state-of-the art. As will be shown below, efforts are almost exclusively focused on the prevention of autoantibody-positive disease.

## 2 Prevention versus suppression of inflammation

Before evaluating the results of individual trials, some reflection is required on the concept of prevention. In general, three types of prevention are discerned. Primary prevention refers to intervening before health effects occur. For instance, smoking cessation in healthy persons without autoantibody responses might be helpful in preventing the occurrence of RA-related autoantibodies or maturation of autoantibody responses and thereby reduce the risk for RA. Secondary prevention involves efforts to identify diseases at the earliest possible stage and intervene at a risk stage to prevent further progression to full-blown disease. Tertiary prevention consists of disease management post diagnosis to slow or stop severe progression. Key in all these different forms of prevention is that an outcome is stopped from happening.

**Fig. 1:**
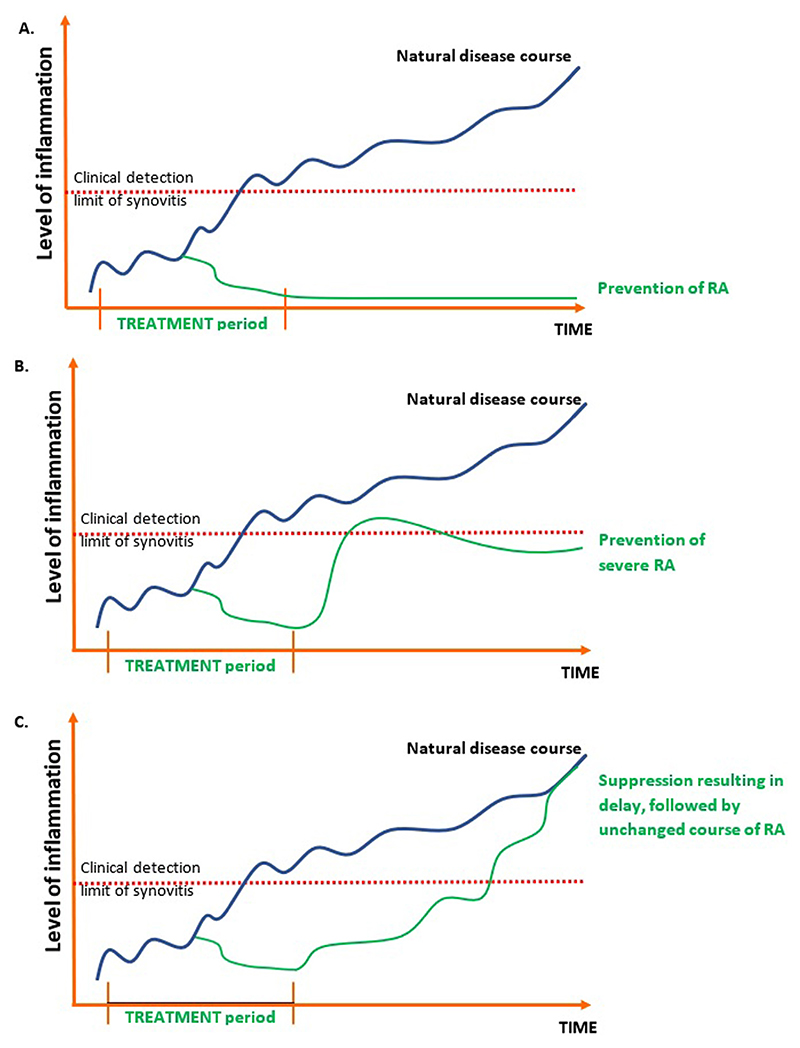
Schematic representation of prevention of inflammation (either of the onset of clinical disease (A) or of severe disease (B)) and suppression of inflammation (C) by temporary treatment in a pre-arthritis phase of RA.

Treatment for secondary prevention in many conditions is continued over time. For instance antihypertensive drugs or statins are continued for decades to prevent cardiovascular events. Preventive efforts should be carefully evaluated on lead time bias. This happens when a disease is earlier detected, before the usual diagnosis, and thereby induces a seemingly prolonged survival. Second prevention in of RA could get rid of this effect when treatment is provided temporary and modification of the disease course is maintained thereafter, also after treatment stop. As such, secondary prevention is intrinsically different from disease suppression. Suppression means a temporary effect during the intervention period that may be lost after the intervention is stopped. Fig. 1 illustrates the conceptual difference between suppression and (secondary) prevention, in terms of (long-term) disease outcomes after the intervention has taken place. Recent preventive studies included symptomatic patients at risk for RA and evaluated effectiveness of a temporary intervention. These trials can therefore be considered secondary prevention studies.

## 3 Outcomes to evaluate efficacy of secondary prevention

The outcomes that are used to measure the achievement of prevention are strongly related to different perspectives. A clinician in daily rheumatological practice may want to prevent the disease RA and measures this by the development of a clinically apparent arthritis (and a diagnosis of RA). Patients, on the other hand, view this differently, they have indicated that they do not find it very relevant whether a rheumatologist can diagnose clinical arthritis at physical joint examination, but evaluate treatment efficacy by the severity of symptoms and functional limitations. In other words, from a patient’s perspective, prevention means reducing existing symptoms and physical limitations as much as possible and preventing further complaints and functional problems in daily life and at work. Immunologists generally have a view from their interest in autoimmunity and often from a research interest in specific subsets of immune cells. As an example of research interest, the maturation of the ACPA response has been extensively studied during the last decades. Current data suggest that maturation of this response occurs 2–3 years before diagnosis: this is reflected by an increase in e.g. number of isotypes and fine-specificities, rise in F(ab)-glycosylation and all of these changes correlate with a rise in ACPA level [3,4]. From this perspective, a preventive intervention can be considered effective if it results in seroconversion from high to normal ACPA levels (i.e. ACPA negativity). When interested in immune cells, a comparable efficacy outcome can be devised (e.g. number of activated B-cells, regulatory T-cells etcetera). Another perspective focuses on imaging, based on the idea that physical examination may not be sensitive enough to detect joint inflammation and that current diagnostics of clinical arthritis and RA are to some extent outdated. It is argued that imaging techniques may be useful in the early detection of joint inflammation and that MRI has been shown to be the most sensitive imaging modality [5]. Subsequently, the effect of preventive interventions can be assessed using imaging detected subclinical joint inflammation.

As will be discussed below, all these different perspectives have been used in the design of the currently available prevention trials. When interpreting these studies, it is important to realize that these outcomes, while related to some extent, cannot be used interchangeably and evaluate intrinsically different processes. For instance, subtle MRI-detected subclinical joint inflammation is present in the healthy population, especially at older age [6]. Moreover true subclinical joint inflammation (“more than the age and location matched reference from the general population”) does not progress to clinical arthritis and RA in a majority of cases (±70%) [7]. Likewise, ACPA can be present in the general population. Even when present in persons with musculoskeletal symptoms (also in high levels), only ±30% of persons develop RA whereas 70% of ACPA-positive individuals with arthralgia do not [8]. If having ACPA or subclinical joint inflammation were considered equivalent to having RA, this leads to an apparent significant increase in the incidence of the disease. Not to mention the converse that 40-50% of all RA patients never have autoantibodies.

**Fig. 2:**
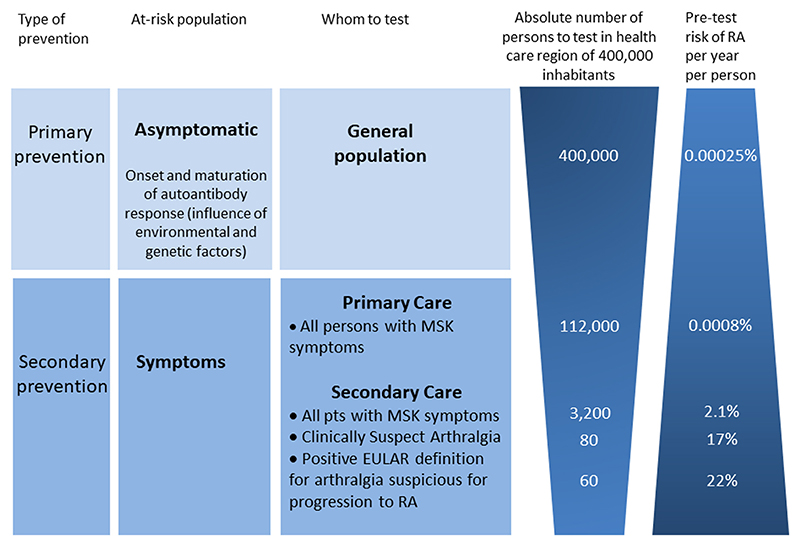
Different approaches to identification of individuals at risk for rheumatoid arthritis correlate with differences in pre-test risk for RA. All numbers in this figure are based on data from the Leiden University Medical Centre (LUMC), the only referral center in a health care region of 400,000 inhabitants, and for some calculations combined with data from the local general practitioner (GP) network practices in this health care region (RNUH-LEO) [9]. In the total general population where the incidence of RA is 25/100,000 per year [10]. Secondary prevention can be performed in arthralgia patients in different health care settings. In primary care, according to NIVEL and the local GP network practices [9,11], the yearly incidence of any non-traumatic musculoskeletal symptom was 294/1,000 (based on ICPC codes L1-L20, L84-L93 and T92 in the period 2009–2013). In a region of 400,000 inhabitants, there will be thus approximately ~112,000 novel consultations for MSK symptoms per year in primary care. Calculations on the yearly risk on RA within this group were based on published data from local GP network practices in the referral region of the LUMC [9]. The total population in this study was 44,350 patients and based on the incidence of consultations for MSK symptoms, it is estimated that approximately 13,000 consultations for MSK complaints were performed in the GP practices yearly. During 2009–2013, 43 polyarthritis cases and 8 oligoarthritis cases were observed and confirmed [12]. Thus, an incidence of 10.2 per year per 13,000 MSK complaints consultations. This is a yearly risk of 0.0008% for the patients presenting with MSK symptoms in primary care. In secondary care, 3200 novel referred patients are seen per year at the rheumatologic outpatient clinic of the LUMC and we assumed that they all have MSK symptoms. Of these, 70 were newly diagnosed with RA within the first year (average data, based on data of the Leiden Early Arthritis Cohort of the period 2009–2013 [13]. Thus, yearly risk of 70/3200 = 2.1%. At this outpatient clinic, 145 CSA-patients were identified in 1.8 year [14]; this is 80 per year. 75% of these CSA-patients had a positive EULAR definition and 22% progressed to RA [15]. As a reference, of all CSA-patients 17% progressed to RA within one year [7].

Given the relationships between these outcomes but the absence of interchangeability, it can be concluded that the effect of preventive studies can be measured with either the occurrence of the disease itself (clinically apparent persistent arthritis/RA) or with an effect on the occurrence or severity of risk factors for RA (e.g. autoantibodies, subclinical joint inflammation), as illustrated in Fig. 2.

## 4 Lessons from trials in Undifferentiated Arthritis

In the early years of 2000 the first secondary prevention trials were done, treating patients with clinically apparent but undifferentiated arthritis (UA), aiming to prevent progression to RA. Irrespective of the medication used in these trials (methylprednisolone im, methotrexate, infliximab, abatacept), the onset of RA was not prevented [16]. Whilst a delay in onset has been reported during methotrexate treatment, the difference got lost after treatment stop, suggestive for a suppressive rather than preventive effect [17]. Interestingly, a meta-analysis of these trials did show a preventive effect [18]. Because the individual trials were relatively small in sample size, the finding of this meta-analysis may touch on the difficulty of properly powering prevention trials.

## 5 The clinicostatistical tragedy and prevention trials

It is a well-known phenomenon that heterogeneity in patient population and corresponding prognosis can blur treatment effects. Feinstein had referred to this as the clinicostatistical tragedy. He provided some examples of how failure to stratify for prognosis leaves a trial clinically defective as early as 1968 [19]. He provided compelling reasoning why subgroup analyses are important if there are potentially large differences in the risk of an outcome. These sub analyses should be predefined, carefully justified and limited to a few analyses, in order to avoid data dredging [20,21]. However from the clinical point of view, the results from large trials cannot be applied to a general patient in case of heterogeneity in risks of an outcome, and individual characteristics should be considered.

A post-hoc study from the PROMPT trial in UA provides an example from a positive effect that was blurred by heterogeneity in risk of RA in a study population. At the time of the design of the PROMPT trial, the risk to progress from UA to RA was about 30% [17]. Risk prediction or risk stratification was not included in trial design or primary analyses. A post-hoc analysis however used a validated risk prediction method for UA and analyzed only the UA-patients with a prediction score of ≥ 8 (positive predictive value of ≥ 84% for developing RA) and reinvestigated the effect of a 1-year course of MTX during 5 years of follow-up [22]. This showed that 55% of methotrexate-treated patients progressed to RA compared to 100% of placebo-treated patients. Beneficial effects of MTX were observed both in ACPA–positive and in ACPA-negative UA patients with a high risk of RA, but not in UA patients without a high risk of RA [22]. Therefore, the interpretation of the results of the PROMPT trial may be, not that MTX is not effective in UA-patients, but that MTX is not effective in UA-patients without a high risk of developing RA.

## 6 Risk prediction in pre-arthritis stages of RA

**Fig. 3:**
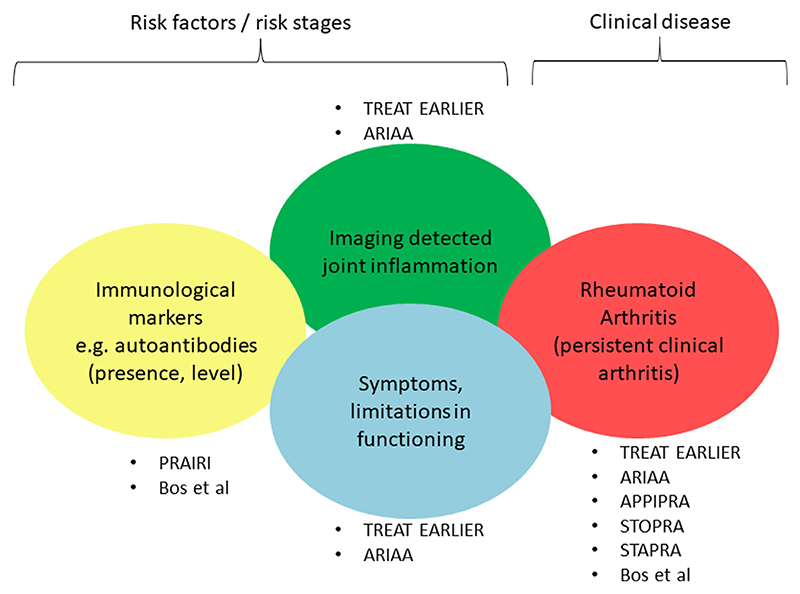
Endpoints reported in prevention trials conducted in the pre-arthritic stage of RA; despite correlations, these cannot be used interchangeably. the different endpoints relate to either development of clinical disease (clinically apparent arthritis/RA) or to risk factors for or risk stages of developing RA. The trials in which the endpoints are used are indicated.

This reasoning and example support the need of developing adequate and validation risk stratification methods for individuals at risk for RA. Positive and negative predictive values strongly depend on prior risks (rule of Bayes). Consequently, high PPV are easier obtained in settings where patients with an increased risk are already selected. Fig. 3 shows the pre-test risk for RA estimated in the general population, in primary care and in secondary care. It increases from 0.00025%/person/year (general population), to 0.008%/person/year (primary care) and about 20%/person/year respectively (CSA patients in secondary care). In clinical practice CSA is identified by clinical expertise and pattern recognition. As this may be somewhat different between rheumatologists, the EULAR definition of arthralgia suspicious for progression to RA was derived by a group of experts to identify homogeneous groups of arthralgia patients. As shown in Fig. 3, using this EULAR definition on top of the clinical expertise provides a small increase in pre-test risk for RA.

As a consequence of difference in pre-test risks, post-test risks vary, also for a test with a high specificity such as ACPA. The PPV of ACPA for RA development in persons from the general population is about 8% [23]. This implies that persons with a positive ACPA test have a false positive test result in 92% of cases. In nonspecific arthralgia in secondary care PPVs of about 30% have been described and in CSA risks of up to 63% have been reported [8,24]. These pre-test risks indicate that high predictive values and accurate predictive tools are easier obtained in patients selected because of CSA than in a symptom-free population (rule of Bayes). Moreover, considering the absolute risks in the light of the “clinicostatistical tragedy” implies that prevention trials more easily show preventive effects in high risk compared to low risk (sub)populations.

Several prediction models have been derived in arthralgia populations [8,25,26]. So far, none of these however is validated in independent studies. Therefor a currently ongoing EULAR taskforce aims to derive validated risk stratification methodology, in order to enable the inclusion of homogenous groups of patients in future prevention trials. This taskforce with expert from all over Europe and the USA started in 2021. Data from > 10 arthralgia cohorts are included and results are awaited in 2023.

## 7 Results from trials in at-risk populations

Table 1 and Table 2 provide an overview of the design and results of performed prevention trials; information was retrieved either from peer reviewed articles or abstracts presented at congresses. Overall, the focus was at prevention of ACPA-positive disease. The outcomes or endpoints used differed between prevention of RA, reduction of ACPA levels, reduction of subclinical joint inflammation and/or improving the disease burden. Also the disease modifying drugs ranged between mild and cheap DMARDs (such as intra-muscular corticosteroid injections and hydroxychloroquine), biologic DMARDs (rituximab and abatacept), and interventions other than DMARDs (atorvastatin and a multidisciplinary lifestyle program). All but one trial was performed in patients with arthralgia visiting secondary care. Only the STOP-RA trial included a mixed population, 63% of ACPA-positive individuals were retrieved from clinics; the remaining part were retrieved from the population.

The STAP-RA trial was prematurely closed due to insufficient inclusions. It was aimed to include 220 patients and stopped after randomization of 62 participants. Analyses of available data suggested no preventive effects for atorvastatin [27]. The Plants-for-Joints trials included a low number of arthralgia patients. The intervention in this trial included plant-based diets, physical activity and stress management in a 16-week life style management program [28,29]. 16 arthralgia patients were included, 14 patients were randomized and after data were studied after 16 weeks the data suggested that the lifestyle program did not influence risk for RA, pain or autoantibody levels. The STOP-RA trial planned to include 200 individuals, treat with hydroxychloroquine for 1 year and follow persons at risk for 3 year on RA development. The study was also prematurely stopped. Interim analyses of 142 included patients demonstrated to benefit from a 1-year course of hydroxychloroquine [30]. Altogether, although the analyzed data may be underpowered, the results obtained suggested no preventive effects for atorvastatin, a multi-disciplinary lifestyle program or hydroxychloroquine.

Of the completed trials, the efficacy results varied. Intramuscular corticosteroids did not prevent RA-development. Although it resulted in a decreased ACPA level, seroconversion to ACPA-negativity was not observed. The PRAIRI trial studied a single gift of rituximab (after methylprednisolone 100 mg intramuscular premedication) and showed a delay but no prevention of RA development.

The TREAT EARLIER trial is the only trial that included autoantibody-positive and autoantibody-negative patients at risk for RA. Inclusion was based on the presence of CSA and a hand and foot MRI positive for subclinical joint inflammation [31,32]. It showed that a single gift of IM corticosteroids and a one year course of methotrexate did not prevent RA-development after 2-years of follow-up. A prespecified sub analysis in high risk CSA patients (those with autoantibodies and subclinical inflammation at multiple locations) showed a difference in RA development during the year of treatment, and also at 18 months follow-up, whilst this was lost after 24 months of follow-up. This demonstrates the need for long-term follow-up to establish true prevention and to rule out that initial findings cannot be attributed to suppressive effects of long-acting DMARDs. Moreover, the key secondary endpoints from the TREAT EARLIER trial were positive. CSA-patients in the methotrexate arm had less severe pain, morning stiffness, limitations in physical functioning and at work compared to the placebo arm. These differences occurred during treatment and persisted during follow-up, also after treatment stop. These findings were in line with the data of MRI-detected joint inflammation. Treatment associated with less severe joint inflammation and this effect was also sustained in the second year without treatment. Since functional disabilities and symptoms in the CSA-phase are reported to be related to the severity of subclinical joint inflammation, the consistency in the results provides face-validity. These data from the TREAT EARLIER trial support the notion that treatment in a pre-disease stage can modify the disease course. Interestingly, persistent reductions in imaging-detected joint inflammation and symptoms were present in both ACPA-positive and ACPA-negative CSA-patients. This is important since the TREAT EALIER trial is the only trial to include autoantibody-negative patients and these results suggest that very early intervention might also benefit autoantibody-negative patients at risk for RA.

The ARIAA trial, as retrieved from congress abstracts, showed a lower frequency of RA development in ACPA-positive arthralgia patients temporarily treated with abatacept: 35% progressed to RA compared to 57% in the placebo arm (*P* = 0.04) at 18 months follow-up [33]. A 24-month measurement was not included. During the first 1.5 years, differences were also present for MRI detected joint inflammation. The APPIPRA trial has a roughly similar design as the ARIAA and also used abatacept [34]. These results are to be awaited.

## 8 Summary of current findings from trials in at-risk populations

In sum, convincing data showing prevention of RA by intervention in the symptomatic risk stage has not yet been provided. Although some efficacy results differed, a clear relation between the type of treatment (conventional versus biologic DMARD) was not observed as hydroxychloroquine on the one hand (mild cvD-MARD) and rituximab on the other hand (bDMARD) did not induce prevention. The full text articles showing the results of the trials using abatacept are awaited.

The patient groups included in the different trials varied slightly but the main denominator was the presence of autoantibodies and of symptoms. Only one study included autoantibody-negative CSA patients and did not suggest that efficacy is different between autoantibody-positive and -negative participants as in both disease subsets patients treated with methotrexate had sustained reductions in joint inflammation, symptoms an disabilities.

## 9 Findings of RA prevention from animal studies

**Fig. 4:**
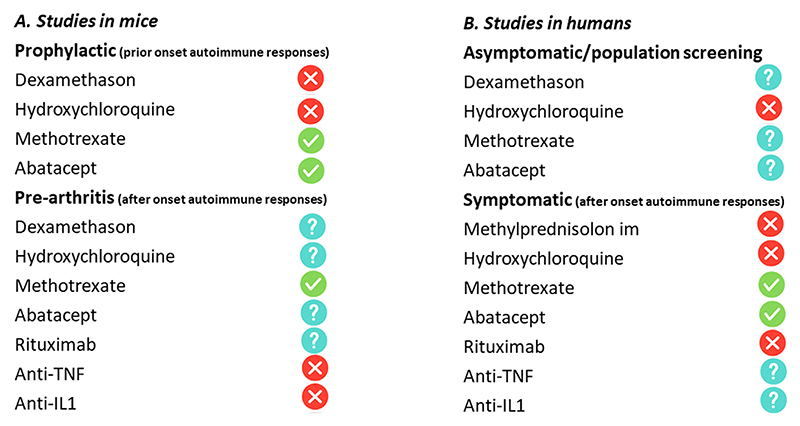
Sustained preventive effects on inflammation (either imaging-detected joint or clinically apparent joint inflammation) of different pre-arthritis treatments in mice (A) and humans (B). Cross: no positive effect observed. Finch: at least one study with a positive effect. Question mark: not studied.

While performing prevention trials in humans is enormously time-consuming, animal studies are more easily done. A meta-analysis summarized the results from arthritis prevention obtained in mice [35]. Prophylactic treatment was differentiated from pre-arthritis treatment. In prophylactic treatment: therapy was initiated prior to injection of arthritis stimulators or after injection of arthritis stimulator but prior to the development of a systemic autoimmune response. Pre-arthritis treatment started after the development of autoimmunity but before the onset of clinically evident arthritis. Prophylactic treatment was effective with methotrexate and abatacept, but not hydroxychloroquine and dexamethasone (Fig. 4). Pre-arthritis treatment was also effective for methotrexate, but not for anti-TNF and anti-IL1. Abatacept and rituximab were not studied. Some quality issues with the design of some studies included in this meta-analyses remain, for instance in some studies it remained unclear whether the treatment was temporarily or sustained until and in the phase of clinical disease. Nonetheless, there is a parallel with the results from the clinical trials. In both mice and humans, effectiveness, if any, appears to be related to interventions involving methotrexate or abatacept (Fig. 4).

## 10 Perspectives and future directions

The trials discussed here represent just the starting point of a new era. Novel trials will follow. Adequate risk stratification is mandatory to prevent false-negative trial results or to reduce the sample size that is required. The results from the EULAR taskforce that derived risk stratification methodology in order to include homogenous groups of persons at risk for RA are urgently needed for accurate trial design. The type of intervention to be used in subsequent trials remains a crucial issue. Importantly, the pathophysiological mechanism driving the progression from CSA to RA and thus yielding the final hit or step of RA development is still unknown. Consequently, secondary prevention cannot be targeted based on the decisive underlying mechanism. Fundamental studies unraveling these mechanisms are required in order to achieve targeted prevention of RA over time.

The currently available data also leaves room for discussion. Is reduction of disease burden a sufficient reason to start DMARDs in patients with CSA and subclinical inflammation? Sustained treatments effects were present in patients that progressed to RA and also in patients that did not develop RA [32]. Hence, overtreatment might not be considered a threat here because effects were not confined to one subgroup. Nonetheless symptom and disability reduction in the “pre-RA field” is a novel era. The presence of more trials showing beneficial effects would be the first requirement towards guidelines on treatment start in pre-disease stages. In addition, the effect sizes that are considered useful and are cost-effective need to be determined.

When treatment initiation in the phase of CSA would be considered, an equally important question is when and how treatment should be stopped. Since treatment with e.g. methotrexate or rituximab was effective during treatment (but differences disappeared 1 year after treatment stop), it has been suggested that interventions in pre-RA should not be temporary but should be continued. The dilemma here is that the starting point for an earlier DMARD start is a shorter disease course, and therefore also an earlier stop. The risk of “not stopping” is that an earlier DMARD start prolongs the course of the disease. Methods for monitoring inflammatory activity at the stage of CSA would be useful. The DAS does not apply to CSA patients because swollen joints are by definition absent; pain alone appears to be insufficiently discriminatory for treatment monitoring. Especially if the pain persists, ways of monitoring subclinical inflammation are warranted to avoid cycling to (expensive) DMARDs. Earlier treatment start without clear plans about treatment discontinuation, harbors the risk of overtreatment. Ideally, the first treatment effects are assessed after 3–4 months of treatment. If effective, treatment can be continued, e.g. up to 1 year, after which it is stopped and restarted only in patients who develop clinical arthritis/RA. Whether such a treatment regimen would be cost-effective and prevent overtreatment remains to be investigated.

Moreover, more attention is need for preventive trials in autoantibody-negative RA. From a pathophysiological view, autoantibody responses are already matured at CSA onset in in autoantibody-positive patients with arthralgia. This might imply that establishing prevention with interventions in the symptomatic at-risk stage is intrinsically difficult. It remains to be investigated whether disease processes can be more easily modified with interventions in autoantibody-negative patients with arthralgia at risk for RA. This would require accurate risk stratification, including in autoantibody-negative individuals at risk for RA.

## 11 Conclusion

In conclusion, the recently published prevention trials in RA are landmark studies in providing proof-of-concept for the treatment of pre-arthritic stage RA and in opening a new era of treatment. However, complete prevention of RA remains elusive. Progress seems to come with small steps rather than with seven-league boots. More small steps, additional data and many discussion, also with patient partners, are needed before results can be summarized in treatment guidelines and implemented in daily rheumatology practice.

## Figures and Tables

**Table 1:** Performed placebo-controlled proof-of-concept studies in a pre-arthritis phase of RA (“preventive trials”).

Trial	Setting	Subjects	N of subjects	Premature closure Y/N	Intervention	Outcome measures
Bos et al 2010	Symptomatic	Arthralgia and ACPA- and/or RF positive, and SE-positive	83	N	Dexamethasone 100 mg intramuscular at 0 and 6 weeks	– 50% reduction or normalisation of autoantibody levels at 6 months^b^ – Development of clinical arthritis^c^
PRAIRI 2016	Symptomatic and Asymptomatic	Arthralgia and ACPA- and RF-positive, and CRP level ≥3 mg/l and/or subclinical synovitis on ultrasound or MRI of the hands	82	Y^a^	Rituximab 1000 mg after methylprednisolone 100 mg intramuscular premedication	– Development of clinically manifest arthritis over mean 29 months^b^
STAPRA 2020	Symptomatic	Arthralgia and ACPA>3xULN or +RF-positivity	62	Y^a^	Atorvastatin 40 mg daily for 3 years	– Development of clinical arthritis^b^
TREAT EARLIER 2022	Symptomatic	Clinically suspect arthralgia with subclinical MRI-inflammation in hand/foot	236	N	Methylprednisolone 120 mg intramuscular once and methotrexate 25 mg during 12 months	– Development of persistent clinically detectable arthritis at 24 months^b^ – Symptoms, disability, workability and MRI detected joint inflammation at 24 months^c^
STOPRA 2022	Asymptomatic and symptomatic	ACPA (anti-CCP3) positivity	144	Y^a^	Hydroxychloroquine 200-400 mg daily during 12 months	– Development of inflammatory arthritis classified as RA^b^
ARIAA 2022	Symptomatic	Arthralgia and ACPA-positive with subclinical MRI-inflammation in the dominant hand	139	N	Abatacept 125 mg sc weekly for 6 months	– Improvement of MRI-inflammation at 6 months^b^ – Progression to RA at 6 and 18 months^c^
APPIPRA	Symptomatic	Non-traumatic arthralgia and auto-antibody positive (RF and ACPA or only high ACPA(>3xULN))	206 planned	N	Abatacept 125 mg sc weekly during 12 months	– Development of clinical arthritis of ≥ 3 joints or RA according to 2010-criteria^b^
Plants for Joint 2022	Symptomatic	ACPA-positive arthralgia	14	NA	Multidisciplinary intervention program (plant based diets, physical activity, stress management) for 16 weeks	– Risk of development of RA (by a risk score)^b^ – Self-reported pain^c^ – ACPA, RF levels at week 16^c^

Information on the STOPRA, STAPRA, Plant for Joints and ARIAA trial were obtained from congress abstracts. Information on the Bos et al, PRAIRI and TREAT EARLIER trial were obtained from peer reviewed articles. Information of the APPIPRA was obtained from trial registry. NA not applicable, it concerned a pilot, planned for 16 participants

a The PRAIRI trial stopped including after 81 randomized patient (90 planned): the STAPRA stopped after randomizing 62 patients (220 planned) and the STOPRA trial stopped after an interim analyses of 144 patients, while 200 inclusions were planned.

b Primary endpoint.

c Other endpoints.

**Table 2:** Summary of results of placebo-controlled proof-of-concept studies in a pre-arthritis phase of RA (“preventive trials”).

Trial	Intervention	% of clinical arthritis development in placebo group (“natural course”)	Findings
Bos et al 2010	Dexamethasone 100 mg intramuscular at 0 and 6 weeks	19.5% after 26 months	– 50% reduction of autoantibody levels achieved in both treatment arms^a^ – No difference in development of clinical arthritis^b^
PRAIRI 2016	Rituximab 1000 mg after methylprednisolone 100 mg intramuscular premedication	40% after 29 months	– No difference in development of clinically manifest arthritis after 29 months^a^ – Delay in developing clinical arthritis in treatment group^b^ – No difference in IgG ACPA, IgG RF between groups, drop in IgM RF in treatment group^b^
STAPRA 2020	Atorvastatin 40 mg daily for 3 years	19% after 18 months	– No difference in development of clinical arthritis^a^
TREAT EARLIER 2022	Methylprednisolone 120 mg intramuscular once and methotrexate 25 mg during 12 months	18% after 24 months	– No difference in development of persistent clinically detectable arthritis at 24 months^a^ – Delay of development of clinical arthritis in high risk group^b^ – Sustained improvements in pain, morning stiffness, physical functioning, presenteeism also in year 2.^b^ – Sustained reduction in MRI detected joint inflammation also in year 2^b^
STOPRA 2022	Hydroxychloroquine 200–400 mg daily during 12 months	36%	– No difference in risk of development of inflammatory arthritis classified as RA^a^
ARIAA 2022	Abatacept 125 mg sc weekly for 6 months	57% after 18 months	– Improvement of MRI-inflammation at 6 months^a^ – Less progression to RA at 18 months (35% vs. 57%)^b^
Plants for Joint 2022	Multidisciplinary intervention program (plant based diets, physical activity, stress management) for 16 weeks	14% in 16 weeks	– No difference in risk of RA development by a risk score^a^ – No difference in self-reported pain, ACPA, RF levels at week 16^b^

Results from the STOPRA, STAPRA, Plant for Joints and ARIAA trial were obtained from congress abstracts. Results from the Bos et al, PRAIRI and TREAT EARLIER trial were obtained from peer reviewed articles. Results from the APPIPRA trial was not yet available.

a Results of primary endpoint.

b Results of other endpoints.
